# Role of drug-induced sleep endoscopy in evaluation of positional vs non-positional OSA

**DOI:** 10.1186/s40463-020-00478-7

**Published:** 2020-12-14

**Authors:** Ming-Chin Lan, Stanley Yung-Chuan Liu, Ming-Ying Lan, Yun-Chen Huang, Tung-Tsun Huang, Yen-Bin Hsu

**Affiliations:** 1 Department of Otolaryngology-Head & Neck Surgery, Taipei Tzu Chi Hospital, Buddhist Tzu Chi Medical Foundation, New Taipei City, Taiwan; 2 School of Medicine, Tzu Chi University, Hualien, Taiwan; 3 Division of Sleep Surgery, Department of Otolaryngology, Stanford University School of Medicine, Stanford, CA USA; 4 Department of Otolaryngology-Head & Neck Surgery, Taipei Veterans General Hospital, No.201, Sec. 2, Shipai Rd., Beitou District, Taipei City, 11217 Taiwan, R.O.C.; 5 School of Medicine, National Yang-Ming University, Taipei, Taiwan

**Keywords:** Obstructive sleep apnea, Positional OSA, Non-positional OSA, Sleep position, Positional dependency, Non-positional dependency, Drug-induced sleep endoscopy

## Abstract

**Background:**

The study aimed to evaluate the anatomical differences between positional and non-positional OSA, and to identify the potential predictors for distinguishing between these two types of OSA.

**Methods:**

A cross-sectional study of 230 consecutive patients with OSA undergoing DISE (Drug-induced Sleep Endoscopy) was carried out at a tertiary academic medical center. The factors correlating with positional and non-positional OSA were analyzed, including clinical characteristics, polysomnography data, and DISE findings.

**Results:**

Univariate analysis revealed that non-positional dependency was correlated with a higher BMI (*p* < 0.001), neck circumference (*p* < 0.001), modified Mallampati score (*p* = 0.003), AHI (*p* < 0.001), degree of velum concentric collapse (*p* = 0.004), degree of oropharyngeal lateral wall collapse (*p* < 0.001), and degree of tongue base anteroposterior collapse (*p* = 0.004). Multivariate analysis revealed that oropharyngeal lateral wall collapse (OR = 1.90, *p* = 0.027) was the only anatomical factor significantly predicted non-positional dependency in OSA patients. AHI (OR = 1.04, *p* < 0.001), although significant, made only a marginal contribution to the prediction of non-positional dependency.

**Conclusions:**

Oropharyngeal lateral wall collapse was identified as the only anatomical predictor for non-positional dependency in OSA patients. Therefore, further treatment modalities should address the distinct anatomical trait between positional and non-positional OSA.

**Graphical abstract:**

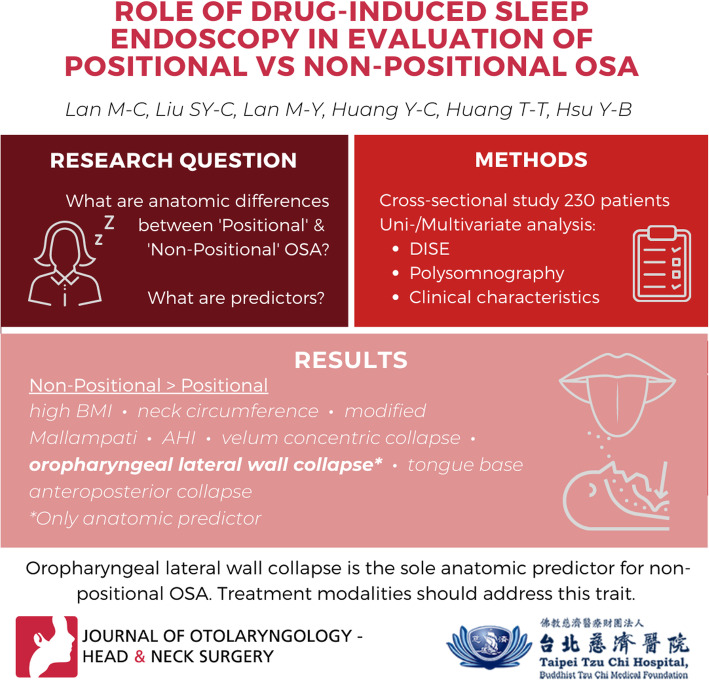

## Introduction

Obstructive sleep apnea (OSA) is a complex disorder with repeated upper airway obstruction during sleep, which results from an interaction between structurally vulnerable anatomy, compromised muscle responsiveness, low respiratory arousal threshold, and unstable ventilatory control [1].

The influence of sleep position on the severity of OSA is possibly related to the effect of gravity. The gravitational effect on the unstable upper airway exacerbates in the supine position, while the detrimental effect of gravity on the upper airway reduces in the lateral decubitus position. Positional OSA, first describe by Cartwright et al., was defined as a supine apnea-hypopnea index (AHI) at least two times greater than a non-supine AHI [2]. A majority of OSA population consists of positional OSA. The prevalence of positional OSA was ranging around 50 to 70%, and the prevalence was even higher in mild-moderate OSA than in severe OSA [3–6].

Several potential factors, including anthropometric measurement, clinical characteristics, and polysomnographic parameters, have been identified to help in differentiating between positional and non-positional OSA. Although there were some discrepancies regarding the difference between positional and non-positional OSA, most of the studies found that positional OSA tends to be younger, non-obese and are associated with mild-moderate OSA rather than severe OSA [3, 7, 8].

Treatment modalities of OSAS are mainly composed of continuous positive airway pressure (CPAP), mandibular advancement devices (MAD), and upper airway surgery. CPAP is typically recommended as gold standard therapy for OSA. However, in well-selected patients, surgery can provide a superior treatment outcome without considering the issue of treatment adherence [9]. Previous studies have indicated that treatment outcomes may vary between positional and non-positional OSA. Weight loss was associated with a greater reduction in non-supine AHI compared to supine AHI [10], whereas positional therapy, MAD and relocation pharyngoplasty resulted in a greater improvement in positional OSA compared with non-positional OSA [11, 12]. Therefore, elucidating the possible mechanism leading to positional vs non-positional dependency could improve the development of personalized management for OSA patients. Drug-induced sleep endoscopy (DISE) is a well-established technique used to evaluate upper airway under unconscious sedation [13]. DISE findings are recorded based on level, pattern and degree of collapse. In this study, DISE was utilized to identify the potential anatomical trait which may account for positional dependency in OSA patients.

The mechanisms underlying positional and non-positional OSA are poorly understood, which might be attributable to distinct clinical characteristics and upper airway anatomy. The aim of this study is to investigate the differences between positional and non-positional OSA, and to identify the potential predictors for distinguishing between these two types of OSA .

## Methods

### Study design and participants

From August 2016 to April 2019, 230 patients were recruited in this cross-sectional study. The inclusion criteria were as follows: (1) age higher than 18; (2) an AHI greater than 5 with OSA related symptoms; and (3) in-lab polysomnography with at least 30 min in supine and non-supine positions. Patients were excluded if they had (1) American Society of Anesthesiologists (ASA) score IV or higher; (2) previous upper airway surgery; (3) neurodegenerative diseases; or (4) congenital anomalies. Patients were classified as positional OSA if they had an overall AHI ≥5 and a ratio of supine AHI/non-supine AHI ≥2 according to the criteria defined by Cartwright [2]. This study was approved by the Institutional Review Board of Taipei Tzu Chi Hospital (IRB No: 05-X06–014).

### Polysomnography study

Standard overnight type I polysomnography (PSG) was performed by trained sleep technicians at the sleep lab. Electroencephalography (EEG), electrooculography, electromyography, electrocardiography, nasal and oral airflow, thoracoabdominal movements, positions, snoring, and oxygen saturation were recorded. The severity of OSA was represented by the apnea hypopnea index (AHI), which was defined as the number of apneas plus hypopneas recorded during the study per hour of sleep. Apneas were defined when the peak signal excursions decreased by more than 90% of the pre-event baseline for at least 10 s. Hypopneas were defined when the peak signal excursions decreased by more than 30% of the pre-event baseline for at least 10 s and associated with more than 3% oxygen desaturation or an EEG arousal. The oxygen desaturation index (ODI) was the number of ≥3% oxygen desaturation episodes recorded during the study per hour of sleep.

### Drug-induced sleep endoscopy and VOTE classification

Patients underwent DISE in a dim operating room, while anesthesiologists carefully monitored oxygenation and circulation during the examination. Sleep induction was maintained with propofol by using a target-controlled infusion (TCI) system. The depth of anesthesia was assessed by the bispectral index (BIS) with the ideal sedation level of BIS score defined between 50 to 70. DISE findings were recorded by VOTE classification. The levels are classified as the velum, oropharynx, base of tongue, or epiglottis. The configuration is defined as anteroposterior, lateral, or concentric. The degree of obstruction is graded as no obstruction (0, no vibration, < 50% obstruction), partial obstruction (1, vibration, 50–75% obstruction), and total obstruction (2, > 75% obstruction) [14].

### Statistical analysis

Descriptive statistics were described as mean ± standard deviation. The difference of demographic data between positional and non-positional OSA was compared using the Mann-Whitney U test in a significant level set at *p* < 0.05. Associations between collapse patterns and positional/non-positional OSA were analyzed using the chi-square test and Fisher’s exact test. Univariate and multivariate logistic regression analyses were used to assess potential factors associated with positional/non-positional dependency in patients with OSA. All Statistical analyses were performed using SPSS version 20.0 (IBM Corp., Armonk, NY).

## Results

The study population consisted of 230 patients. Among these, 174 (75.7%) were positional OSA, while 56 (24.3%) were non-positional OSA. The mean AHI was 36.52 ± 23.44 events/hour, and the mean body mass index (BMI) was 27.50 ± 4.33 kg/m^2^.

Nonparametric Mann-Whitney U test was used to compare the difference of demographic data, clinical features, and sleep study parameters between positional and non-positional OSA. The results showed that BMI (*p* < 0.001), neck circumference (*p* = 0.002), modified Mallampati score (*p* = 0.003), AHI (*p* < 0.001), supine AHI (*p* < 0.001), lateral AHI (*p* < 0.001), ODI (*p* < 0.001), Minimal SaO2 (minimal oxygen saturation, *p* < 0.001), and T < 90% (the percent of the total time with oxygen saturation level lower than 90%, *p* < 0.001) were significantly different between positional and non-positional OSA (Table 1).

**Table 1:** Demographic, physical examination, and sleep study data

Variables	Total (*n* = 230)	Positional OSA (*n* = 174)	Non-positional OSA (*n* = 56)	*P* value*
	Mean ± SD	Mean ± SD	Mean ± SD	
**Demographic data**				
Sex				.219
Male	166 (72.2%)	122 (70.1%)	44 (78.6%)	
Female	64 (27.8%)	52 (29.9%)	12 (21.4%)	
Age, years	48.37 ± 11.50	48.71 ± 11.30	47.34 ± 12.14	.416
BMI, kg/m^2^	27.50 ± 4.33	26.68 ± 3.85	30.05 ± 4.76	< 0.001
Neck circumference, cm	38.26 ± 3.84	37.70 ± 3.47	40.01 ± 4.42	.002
Epworth Sleepiness Scale	9.94 ± 4.53	10.08 ± 4.23	9.52 ± 5.39	.112
**Physical Examination**				
Tonsil Size	1.39 ± 0.69	1.40 ± 0.68	1.38 ± 0.73	.778
Modified Mallampati score	3.16 ± 0.61	3.09 ± 0.62	3.38 ± 0.56	.003
**Sleep Study Data**				
AHI, events/hr	36.52 ± 23.44	29.94 ± 16.96	56.98 ± 28.64	< 0.001
Supine AHI, events/hr	48.36 ± 25.39	43.86 ± 22.47	62.32 ± 28.89	< 0.001
Lateral AHI, events/hr	18.72 ± 24.22	8.15 ± 8.32	51.56 ± 27.82	< 0.001
ODI, events/hr	30.14 ± 23.54	23.16 ± 16.41	51.84 ± 28.76	< 0.001
Minimal SaO_2_, %	77.74 ± 9.67	79.74 ± 8.75	71.52 ± 9.80	< 0.001
T < 90%, %	15.09 ± 18.53	10.03 ± 12.63	30.80 ± 24.41	< 0.001

*BMI* Body Mass Index, *AHI* Apnea-Hypopnea Index, *ODI* Oxygen Desaturation Index, *Minimal SaO*_*2*_ Minimal oxygen saturation, *T < 90%* The percent of the total time with oxygen saturation level lower than 90%

*Mann-Whitney U test

Univariate logistic regression analysis (Table 2) revealed that the statistically significant variables associated with non-positional dependency are BMI (*p* < 0.001), neck circumference (*p* < 0.001), modified Mallampati score (*p* = 0.003), AHI (*p* < 0.001), velum concentric collapse (*p* = 0.004), oropharyngeal lateral wall collapse (*p* < 0.001), and tongue base anteroposterior collapse (*p* = 0.004). The proportion of patients having velum concentric collapse, oropharyngeal lateral wall collapse, or tongue base anteroposterior collapse in positional and non-positional OSA were further displayed in Tables 3, 4 and 5.

**Table 2:** Univariate and multivariate logistic regression model to identify factors associated with positional and non-positional obstructive sleep apnea

Variables	Univariate		Multivariate	
	Odds Ratio (95% CI)	*P* value	Odds Ratio (95% CI)	*P* value
**Demographic data**				
Sex	1.56 (0.76–3.20)	.222	0.86 (0.24–3.11)	.819
Age, years	0.99 (0.95–1.02)	.438		
BMI, kg/m2	1.20 (1.11–1.30)	< 0.001	1.11 (0.97–1.28)	.137
Neck circumference, cm	1.18 (1.08–1.29)	< 0.001	0.95 (0.78–1.16	.629
Epworth Sleepiness Scale	0.97 (0.91–1.04)	.419		
**Physical Examination**				
Tonsil Size	0.95 (0.61–1.48)	.822		
Modified Mallampati score	2.20 (1.30–3.73)	.003	1.49 (0.75–2.99)	.258
Retrognathia	1.08 (0.44–2.67)	.867		
**Sleep Study Data**				
AHI, events/hr	1.05 (1.04–1.07)	< 0.001	1.04 (1.03–1.06)	< 0.001
**DISE findings**				
Velum				
A-P	0.70 (0.48–1.02)	.062	1.28 (0.79–2.07)	.310
Concentric	1.74 (1.19–2.56)	.004	0.93 (0.58–1.49)	.767
Oropharynx				
Lateral	2.55 (1.64–3.97)	< 0.001	1.90 (1.08–3.33)	.027
Tongue base				
A-P	1.86 (1.22–2.82)	.004	1.27 (0.75–2.13)	.371
Epiglottis				
A-P	1.30 (0.86–1.97)	.220	1.64 (0.94–2.83)	.079
Lateral	1.46 (0.78–2.70)	.235	1.54 (0.72–3.33)	.268

*BMI* Body Mass Index, *AHI* Apnea-Hypopnea Index, *ODI* Oxygen Desaturation Index, *A-P* Anteroposterior

**Table 3:** Velum concentric collapse in positional and non-positional obstructive sleep apnea

Collapse Pattern			Positional OSA (*n* = 174)	Non-positional OSA (*n* = 56)	*P* value*
Level	Configuration	Degree	n (% within Position OSA)	n (% within Non-position OSA)	
Velum	Concentric	0	64 (36.8%)	10 (17.9%)	0.004
		1	7 (4.0%)	0 (0%)	
		2	103 (59.2%)	46 (82.1%)	

*OSA* Obstructive sleep apnea

*Chi-square test and Fisher’s exact test

**Table 4:** Oropharynx lateral wall collapse in positional and non-positional obstructive sleep apnea

Collapse Pattern			Positional OSA (*n* = 174)	Non-positional OSA (*n* = 56)	*P* value*
Level	Configuration	Degree	n (% within Position OSA)	n (% within Non-position OSA)	
Oropharynx	Lateral	0	58 (33.3%)	5 (8.9%)	< 0.001
		1	59 (33.9%)	16 (28.6%)	
		2	57 (32.8%)	35 (62.5%)	

*OSA* Obstructive sleep apnea

*Chi-squared test

**Table 5:** Tongue base anteroposterior collapse in positional and non-positional obstructive sleep apnea

Collapse Pattern			Positional OSA (*n* = 174)	Non-positional OSA (*n* = 56)	*P* value*
Level	Configuration	Degree	n (% within Position OSA)	n (% within Non-position OSA)	
Tongue Base	A-P	0	54 (31%)	8 (14.3%)	0.003
		1	73 (42%)	23 (41.1%)	
		2	47 (27%)	25 (44.6%)	

*OSA* Obstructive sleep apnea, *A-P* Anteroposterior

*Chi-squared test

The proportion of patients having partial or complete velum concentric collapse was higher in non-positional OSA (82.1%) when compared to positional OSA (63.2%). Pearson’s chi-square test and Fisher’s exact test showed a significant correlation between non-positional dependency and velum concentric collapse during DISE (*p* = 0.004, Table 3).

The proportion of patients having partial or complete oropharyngeal lateral wall collapse was higher in non-positional OSA (91.1%) when compared to positional OSA (66.7%). Pearson’s chi-square test showed a significant correlation between non-positional dependency and oropharyngeal lateral wall collapse during DISE (*p* < 0.001, Table 4). Oropharyngeal lateral wall collapse in DISE findings had high sensitivity (91.1%) and negative predictive value (92.1%) for non-positional OSA. However, the specificity and positive predictive value were relatively low (33.3 and 30.5%, respectively).

The proportion of patients having partial or complete tongue base anteroposterior collapse was higher in non-positional OSA (85.7%) when compared to positional OSA (69%). Pearson’s chi-square test showed a significant correlation between non-positional dependency and tongue base anteroposterior collapse during DISE (*p* = 0.003, Table 5).

After multivariate logistic regression analysis (Table 2), oropharyngeal lateral wall collapse (OR = 1.90, *p* = 0.027) was the only anatomical factor associated with the prediction of non-positional dependency in OSA patients. AHI (OR = 1.04, *p* < 0.001), although significant, added only a marginal contribution to the prediction of non-positional dependency. In summary, severe OSA patients with oropharyngeal lateral wall collapse were significantly more likely to be non-positional than mild-moderate OSA patients without oropharyngeal lateral wall collapse.

## Discussion

Previous studies have identified age, neck circumference, BMI, Mallampati score, and AHI as potential predictors to discriminate between positional and non-positional OSA [3, 7, 8, 15]. However, no previous studies have incorporated DISE findings as potential factors to predict positional/non-positional dependency. In this study, patients with non-positional OSA had higher BMI, neck circumference, modified Mallampati score, AHI and oximetry variables when compared to patients with positional OSA. Besides, patients with non-positional OSA were associated with a higher prevalence of velum concentric collapse, oropharyngeal lateral wall collapse, and tongue base anteroposterior collapse when compared to patients with positional OSA. After multivariate analysis, only AHI and oropharyngeal lateral wall collapse were identified as the statistically significant clinical predictors to determine non-positional dependency in patients with OSA.

Fewer studies have evaluated the role of anatomical structures to discriminate between positional and non-positional OSA. Richard et al. have reported that the proportion of retropalatal obstruction and retrolingual obstruction did not differ significantly between positional and non-positional OSA. However, in their study, the results of sleep endoscopy were simply divided into velum collapse and tongue base collapse, without mention of lateral pharyngeal wall and epiglottis [16]. Victores et al. have indicated that at least a partial improvement of tongue base and epiglottis collapse were observed in nearly all patients with positional OSA while in the lateral sleep position. By contrast, upper airway collapse did not alter significantly between supine and lateral position in patients with non-positional OSA [17]. In a population of mostly nonobese adults, Yalamanchili et al. have showed a greater likelihood of anteroposterior airway narrowing (velum, tongue base, and epiglottis) in the supine position in both positional and non-positional OSA. In non-positional OSA, there was a higher degree of oropharyngeal lateral wall collapse in the lateral position [18]. In the study of Lee et al., they have found that oropharyngeal lateral wall collapse did not show significant improvement after position change (70.6% in supine vs. 60.0% in lateral). Moreover, persisted lateral pharyngeal wall collapse in the lateral position was observed more frequently in non-positional OSA as compared to positional OSA (83.3% vs. 33.3%). Therefore, they concluded that non-positional dependency is mainly determined by collapsibility of lateral pharyngeal wall [19].

The detrimental effect of gravity on the velum, tongue base or epiglottis collapse decreased when the position was shifted from the supine position into the lateral position. As a result, AHI was reduced in the lateral position as compared with the supine position. On the contrary, gravity had limited effect on the lateral pharyngeal wall collapse. Therefore, the lateral pharyngeal wall collapse remained unchanged when the sleep position was shifted from the supine position into the lateral position. Since patients with non-positional OSA had a higher prevalence and increased severity of lateral pharyngeal wall collapse, a less reduction of AHI in the lateral position was expected in patients with non-position OSA.

Schwarts et al. described that the degree of collapse at lateral pharyngeal wall, which was evaluated by endoscopic Mueller maneuver, correlated significantly with OSA severity [20]. Similarly, previous research utilizing DISE demonstrated a strong correlation between oropharyngeal lateral wall collapse and OSA severity [21]. In this study, patients with non-positional OSA had a higher prevalence of lateral oropharyngeal wall collapse. Therefore, it seems logical that patients with non-positional OSA are associated with increased severity of OSA. Several treatment options have been proposed to alleviate the symptoms of OSA. However, the response rate of each treatment modality varied between positional and non-positional OSA. The distinct anatomical trait between positional and non-positional OSA could partially illustrate the different therapeutic effects between positional and non-positional OSA, as further described below. First, Joosten et al. found that weight loss resulted in a greater reduction in non-supine AHI than supine AHI [10]. Since higher BMI is associated with a higher degree of oropharyngeal lateral wall collapse [21], a decrease in BMI may lessen the severity of oropharyngeal lateral wall collapse, which in turn leads to a reduction in non-supine AHI. Second, a multicenter French cohort revealed that patients with positional OSA had a significantly lower likelihood of CPAP adherence and an increased risk of CPAP withdrawal as compared with non- positional OSA [22]. Previous studies utilized DISE to evaluate upper airway structural changes induced by CPAP titration [23, 24]. They concluded that the therapeutic effect of CPAP treatment on upper airway collapse seemed mainly to be mediated through oropharyngeal lateral wall. As a result, a greater improvement in lateral collapse rather than anterior-posterior collapse was observed [23], which indirectly explained the higher CPAP adherence in patients with non-positional OSA. Third, post-maxillomandibular advancement (MMA) improvement of upper airway collapse was most predominant at the level of oropharyngeal lateral wall [25]. Therefore, the surgical success of total and non-supine AHI was significantly greater in non-positional OSA as compared with positional OSA undergoing MMA [26]. Fourth, positional OSA has been considered to possess a more favorable outcome in MAD when compared with non-positional OSA in the literature [11]. A previous study showed that crowding of the oropharynx was thought to be one of the clinical features associated with treatment deterioration in MAD [27]. Therefore, positional OSA has been associated with better treatment outcomes in MAD. Lastly, Li et al. have shown that patients with non-positional OSA had a significantly lower surgical success rate than patients with positional OSA undergoing relocation pharyngoplasty [12]. Besides, Soares et al. have indicated that the presence of severe lateral pharyngeal wall collapse on preoperative DISE was associated with a higher surgical failure rate [28]. Therefore, the lower surgical success rate of non-positional OSA may result from the presence of lateral pharyngeal wall collapse. In summary, treatment strategies addressing oropharyngeal lateral wall, such as weight loss, CPAP, and MMA, led to better treatment outcomes in non-positional OSA. On the other hand, treatment strategies, including positional therapy, MAD, and relocation pharyngoplasty, had preferable success rates in positional OSA. Due to the distinct anatomical trait between positional and non-positional OSA, further treatment modalities should be tailored accordingly.

### Limitations

This study has several limitations. First, the pathophysiology of positional dependency may not be fully illustrated by anatomic traits. Since OSA is a complex and heterogeneous disorder, non-anatomic traits, such as low respiratory arousal threshold, unstable ventilatory control, and ineffective pharyngeal dilator muscle activity, may also differ between positional and non-positional OSA [1]. Second, DISE is performed under propofol sedation, the results may not truly reflect the genuine condition during natural sleep. However, previous research has indicated that collapse patterns of the upper airway appeared to be in agreement between DISE and natural sleep endoscopy. It seems therefore that DISE is a relatively reliable exam [29]. Third, the diagnosis of positional dependency and the prescription of positional therapy are fundamentally based on PSG reports rather than DISE findings, although DISE could better elucidate the underlying anatomical dissimilarities between positional and non-positional OSA. Last, although type I PSG provides more comprehensive reports as compared with home sleep testing, multiple channels with type I PSG might restrict body movements and predispose to more supine sleep. As a result, the AHI may be overestimated, especially in positional OSA [30].

## Conclusion

In this study, patients with non-positional OSA had higher BMI, neck circumference, modified Mallampati score, AHI and ODI oximetry variables when compared to patients with positional OSA. Besides, a higher prevalence of velum concentric collapse, oropharyngeal lateral wall collapse, and tongue base anteroposterior collapse were observed in patients with non-positional OSA. However, after multivariate analysis, oropharyngeal lateral wall collapse was the only anatomical predictor for non-positional dependency in OSA patients. Therefore, further treatment strategies should be tailored based on the distinct anatomical trait between positional and non-positional OSA.

## Data Availability

All data generated or analyzed during the present study are included in this published article.

## Abbreviations

AASM: American Academy of Sleep Medicine
AHI: Apnea-hypopnea index
ASA: American Society of Anesthesiologists
BIS: Bispectral index
BMI: Body mass index
CPAP: Continuous positive airway pressure
DISE: Drug-induced sleep endoscopy
EEG: Electroencephalography
MAD: Mandibular advancement device
Minimal SaO2: Minimal oxygen saturation
ODI: Oxygen desaturation index
OSA: Obstructive sleep apnea
PSG: Polysomnography
T < 90%: The percent of the total time with oxygen saturation level lower than 90%
TCI: Target-controlled infusion
